# Engineering *Yarrowia lipolytica* for the Synthesis of Glutathione from Organic By-Products

**DOI:** 10.3390/microorganisms8040611

**Published:** 2020-04-23

**Authors:** Diem T. H. Do, Patrick Fickers

**Affiliations:** Microbial Processes and Interactions, TERRA Teaching and Research Center, University of Liège - Gembloux Agro-Bio Tech, Av. de la Faculté, 2B, 5030 Gembloux, Belgium; dthdiem@doct.uliege.be

**Keywords:** glutathione, inulin, *Yarrowia lipolytica*, *GSH1*, *GSH*2, *INU1*, overexpression, bioreactor

## Abstract

Tripeptide glutathione, which plays important roles in many cellular mechanisms, is also a biotechnology-oriented molecule with applications in medicine, food and cosmetic. Here, the engineering of the yeast *Yarrowia lipolytica* for the production of this metabolite at high titer values from various agro-industrial by-products is reported. The constitutive overexpression of the glutathione biosynthetic genes *GSH1* and *GSH2* encoding respectively γ-glutamylcysteine synthetase and glutathione synthetase, together with the *INU1* gene from *Kluyveromyces marxianus* encoding inulinase yielded a glutathione titer value and a productivity of 644 nmol/mg protein and 510 µmol/g_DCW_, respectively. These values were obtained during bioreactor batch cultures in a medium exclusively comprising an extract of Jerusalem artichoke tuber, used as a source of inulin, and ammonium sulfate, used as a nitrogen source.

## 1. Introduction

Tripeptide glutathione (γ-l-glutamyl-l-cysteinyl-glycine, GSH) is present in most living cells. It is a powerful antioxidant that contributes to the intracellular redox balance by maintaining sulfhydryl groups of proteins in a reduced state (see reviews in [1,2]). Beside this, GSH plays a key role in the stress response triggered by nutrient starvation, heavy metals, reactive oxygen species and xenobiotics [1]. Glutathione is also a biotechnology-oriented molecule which is used in medicine as an anti-cancer and anti-aging drug, as well as in the food and beverage industries as an antioxidant or in cosmetics due to its skin-whitening effect [2]. The global GSH market was valued at around 0.8 billion USD in 2017 and is forecasted to reach 2.5 billion USD in 2027, expanding at a CAGR closed to 12% (https://www.researchnester.com/reports/glutathione-market/1157). In *Saccharomyces cerevisiae* and other glutathione-containing organisms, GSH is synthesized in a two-step pathway, where glutamate and cysteine are first linked by γ-glutamylcysteine synthetase (EC6322, encoded by *GSH1*) to form a γ-glutamyl-cysteine dipeptide. In a second step, glycine is linked to the dipeptide by glutathione synthetase (EC6323, encoded by *GSH2*). In cells, GSH is maintained in a reduced state by the activity of a NADPH-dependent glutathione reductase [1]. The biotechnological production of GSH can be performed either by enzymatic reactions with purified enzymes or by fermentation using food-grade microorganisms, such as *S. cerevisiae*. In the latter case, optimizations of both producing strains by evolutionary or metabolic engineering and bioreactor process development have been considered. Genome modifications have consisted, in most cases, of overexpressing GSH biosynthetic genes [2]. Recently, we demonstrated that the overexpression of the gene *MET4* coding for the central regulator of sulfur metabolism in *Ogataea polymorpha* yielded a five-fold increased GSH titer value [3]. However, the productivity of the strains and processes developed to date remain too low for cost-effective production. One reason for this is the high cost of the culture media, which are most often based on pure glucose. Only a few processes have considered industrial by-products such as sugarcane molasses as alternative carbon sources [2,4]. 

The yeast *Yarrowia lipolytica* is known for its high tolerance to heavy metals [5,6], and since this tolerance has been linked to GSH synthesis [1], we hypothesized here that *Y. lipolytica* could be a promising organism for GSH production. Moreover, this yeast is also considered to be a promising cell factory for the conversion of industrial by-products into compounds of biotechnological interest [7,8]. Recent metabolic engineering strategies have been developed to expand the capability of *Y. lipolytica* to hydrolyze di- and polysaccharides (i.e., sucrose, inulin or starch) or to improve the endogenous metabolism of naturally consumed carbon sources (i.e., glucose, glycerol, fructose and hydrophobic substrates) [7,9,10]. 

The worldwide production of organic waste is increasing yearly, leading to serious environmental issues. However, organic waste from various industries is increasingly considered to be a substitution substrate to reduce the process costs [8]. For instance, glycerol—the main byproduct of the biodiesel industry—is used for the synthesis of biofuels and chemicals (for a review, see [11]). Starch, one of the most abundant carbohydrates on earth, is the energy-storage molecule in rice, maize, wheat and potato [12]. It is a complex polymer of glucose formed by amylopectin (α-1, 4-D-glucopyranose chains with α-1,6 branching points) and amylose chains (α-1,4-linked-d-glucopyranose). Waste from bakeries and potato transformation industries contains starch, which could be used as a feedstock. Although they are available in large amounts, these waste products must be processed before their utilization. This occurs by liquefaction using α-amylase, which breaks down the polymer into oligosaccharides, and by saccharification using glucoamylase, which liberates glucose and maltose units from the oligosaccharides [13]. For that purpose, *Y. lipolytica*-engineered strains fitted with rice α-amylase and glucoamylase from *Aspergillus niger* have been constructed. They showed the ability to grow on both soluble starch and raw starch [14]. Inulin is a fructose polymer that accumulates as an energy-storage molecule in plants such as blue agave, chicory roots, Jerusalem artichoke, garlic, asparagus roots and Dahlia tubers [15]. As it is widely used as a sugar substitute, diet fibers and pharmaceutical excipients, the industry of inulin transformation generates inulin-rich by-products that could be used as an alternative carbon source to sustain microorganism growth and metabolism [16,17]. Moreover, the hydrolysis of inulin into fructose monomers is less complex than starch pretreatments. The heterologous expression of inulinase-encoding gene confers yeast the ability to hydrolyze inulin and to metabolize the released fructose directly [18,19,20]. Recently, the direct fermentation of Jerusalem artichoke powder for the production of lactic acid by an engineered strain of *Kluyveromyces marxianus* has been reported [21]. Lactose is also a promising carbon source for bioprocesses. Whey is a lactose-rich waste from dairy industries, with a global production of 165 million tons per year [22]. This disaccharide is composed of galactose and glucose subunits linked by β-1-4 glycosidic bonds. The expression of the gene encoding β-galactosidase endows yeast—such as *Y. lipolytica*—with the ability to grow on lactose [23].

With the ever-growing global population, the management of industrial by-products and waste is an important concern, as they represent an increasing environmental burden. However, some of these by-products, such as organic waste, represent an alternative feedstock to provide cell factories with the building blocks and energy required to produce value-added compounds. Herein, we aimed to valorize these organic wastes as substrates for the synthesis of glutathione. This required the overexpression of the endogenous genes *GSH1* and *GSH2* together with genes that endow *Y. lipolytica* with the ability to metabolize the above-mentioned alternative carbon sources.

## 2. Materials and Methods 

### 2.1. Strains, Media and Culture Conditions

The strains used in this study are listed in Table 1. *Escherichia coli* was grown at 37 °C in Luria–Bertani medium supplemented with ampicillin (100 mg/L) or kanamycin sulfate (50 mg/L) when required. For shake flask cultures, yeasts were grown at 28 °C in YPD (20 g/L Difco bacto peptone, 10 g/L Difco yeast extract, 20 g/L glucose) or in YNB medium (1.7 g/L Difco yeast nitrogen base medium without amino acids and ammonium sulfate, 2 g/L Difco casamino acids, 1 g/L ammonium chlorides, 0.05M phosphate buffer pH 6.8 supplemented with leucine (0.2 g/L) or uracil (0.1 g/L) to meet the requirements of auxotrophs) [24]. The YNB medium contained different pure carbon sources (10 g/L), namely glucose (YNBD), glycerol (YNBG), starch (YNBS), inulin (YNBI) and lactose (YNBL) or raw inulin extract from blue agave (*Agave tequilana*, medium YNBIa), Jerusalem artichoke (*Helianthus tuberosus*, medium YNBIja) or chicory (*Cichorium intybus*, medium YNBc). The media for the bioreactor cultures were the INU medium (10 g/L of Jerusalem artichoke powder) and INUC medium (10 g/L of Jerusalem artichoke powder, 5 g/L (NH_4_)_2_SO_4_, 2.5 g/L yeast extract)_._ For solid media, agar (15 g/L) was added. Shake flask cultures were performed in triplicate on a rotary shaker. Bioreactor cultures were performed for 48 h in duplicate in DASGIP DASbox Mini Bioreactors SR0250ODLS (Eppendorf, Hamburg, Germany). Agitation was set at 600 rpm and aeration at 1 vvm. The pH was automatically adjusted to 6.8 by the addition of 8 M H_3_PO_4_ or 12.5 M NaOH. All cultures were seeded at an initial optical density at 600 nm (OD600) of 0.1, with cells grown for 24 h in YPD medium and washed twice with phosphate buffer (50 mM, pH 6.8). 

### 2.2. General Molecular Techniques, Vectors and Strains Construction 

The standard media and techniques used for *E. coli* and *Y. lipolytica* have been described elsewhere [24,25]. The restriction enzymes, DNA polymerases and ligase were supplied by New England BioLabs Inc. Yeast genomic DNA was prepared as described elsewhere [26]. PCRs were performed using the primers listed in Table S1 (Supplementary Materials). Taq DNA polymerase (Biolabs, New England, USA) was used for cloning and DreamTaq DNA polymerase (Thermo Scientific, Waltham, MA, USA) was used to verify the correctness of the cell genotype. The PCR fragments were purified from the agarose gels using a Monarch^®^ DNA Gel Extraction kit (BioLabs, New England, USA). Plasmids were extracted and purified using the GeneJET plasmid Miniprep Kit (Thermo Scientific). The DNA sequencing was performed by GATC Biotech (https://www.gatc-biotech.com), and the primers were synthesized by Eurogentec (https://secure.eurogentec.com/). BLASTp analysis was performed at https://blast.ncbi.nlm.nih.gov/Blast.cgi.

For their constitutive overexpression, *ylGSH1* and *ylGSH2* genes were cloned in the expression vectors RIP102 (*URA3ex*) and RIP103 (*LEU2ex*) as *BamH*I/*AvrI*I fragments. Prior to this step, genes *ylGSH1* and *ylGSH2,* containing internal *BamH*I and *AvrI*I recognition sequences, respectively, were mutagenized by overlap PCR as described below in order to convert the GGATTC (*BamH*I) into GGATTT and CCTAGG (*AvrI*I) into CCTGGG. These modifications did not introduce any change in the amino acid sequence of the corresponding proteins. The *ylGSH1* gene (*YALI0E30129*) was amplified from *Y. lipolytica* JMY2900 genomic DNA using the primer pair N1/N2. The resulting 1884 bp fragment was cloned into the pGEMT-Easy vector to yield plasmid RIP153. To remove the internal *BamH*I site of the amplicon, PCR was performed using the primer pairs N1/N3 and N2/N4 and RIP153 as a template. After purification, the two overlapping fragments (1739 bp and 194 bp) were pooled and amplified using the primer pair N1/N4. The resulting 1884 bp amplicon was then cloned into the pGEMT-Easy vector to yield plasmid RIP161. After the verification of the correctness of the insert by DNA sequencing, plasmid RIP161 was digested by *BamH*I and *Avr*II, and the resulting fragment was cloned into RIP102 and RIP103 plasmids at the corresponding sites, resulting in plasmids RIP210 and RIP211, respectively (Table 1). 

The *ylGSH2* gene (*YALI0C17831*) was cloned following the same procedure, using the primer pair N5/N8. The internal *Avr*II site was removed by PCR using the primer pairs N5/N7 and N6/N8 and RIP155 as a template. The resulting 387 bp and 1117 bp overlapping amplicons were then used as templates for PCR amplification with the primer pair N5/N8. The resulting 1476 bp fragment was then cloned into the pGEMT-Easy vector to yield plasmid RIP162. After the verification of the correctness of the gene sequence, plasmid RIP162 was digested with *BamH*I and *Avr*II, and the resulting fragment was finally cloned into RIP102 and RIP103 at the corresponding sites. The resulting plasmids were named RIP212 and RIP213, respectively (Table 1). 

For yeast transformation, the expression vectors—namely RIP210, RIP211, RIP212, RIP213, RIP282, RIP287, RIP288, JMP2792, FCP015, RIP196 (Table 1)—were first *Not*1 digested, and the purified expression cassettes were used. Vectors RIP210, RIP211 were used to overexpress *ylGSH2,* RIP212, RIP213 were used to overexpress *ylGSH2*, and vectors RIP282, RIP287, RIP288, JMP2792, FCP015, RIP196 were used to overexpress the genes *INU1*, *glucoAMY*, *alphaAMY*, *GUT1*, *GUT2* and *A.oryGal*, respectively (Table 2). Transformants were plated on YNBG medium supplemented with uracil or leucine when required. Marker rescue was performed with the Cre-lox system as previously described [27]. For the constructed strains, the correctness of the genotype was confirmed by analytical PCR using the primer pairs N15/N16 for the integration of gene expression cassettes and N13/N14 after marker rescue with Cre recombinase (RIP112). qPCRs were performed to confirm the overexpression of the different cloned genes using primers N9 to N12 and N17 to N26 depending on the gene considered (Table S1, Supplementary Materials).

### 2.3. RNA Isolation, Transcription and Other Verifications 

The NucleoSpin RNA kit (Macherey-Nagel, Duren, Germany) was used for RNA isolation. RNA quantification was performed using a Nanodrop 2000 spectrophotometer at a wavelength of 260 nm. The qPCRs were performed using a LunaR Universal One-Step RT-qPCR kit (New England-Biolabs, Ipswich, MA, USA) and the primer pairs listed in Table S1 (Supplementary Materials). The cycles of qPCR were as described elsewhere (Sassi et al., 2016). Gene expression levels *(INU1, g-AMY, α-AMY, GUT1, GUT2, β-GAL,* Table 2*)* were standardized using the expression level of the actin gene (GI:6522909) as a reference (ΔCT method, primers N27 and N28). For *ylGSH1* and *ylGSH2*, the fold difference in gene expression between recombinant and wild-type strains was calculated as described elsewhere [33]. Samples were analyzed in triplicate.

### 2.4. Analytical Methods 

Biomass was determined by the cell dry weight and expressed in gDCW/L. The growth rate was calculated as ΔX/Δt, where X is the biomass concentration (g_DCW_/L) at time t (h). For cell extract preparation, cells from one ml of culture broth were collected by centrifugation at 5000 x *g* at room temperature for 10 min. The cell pellet was washed twice with phosphate buffered saline (PBS, pH 6.8) and re-suspended in 1 mL of the same buffer. The cell suspension was disrupted using a FastPrep-24 instrument (MP Biopmedicals, Eschwege, Germany, 4 × 2-min) with 0.3 g of glass beads (acid-washed, Sigma). Cellular debris were then removed by centrifugation (10,000 × *g* for 10 min at 4 °C). The total protein concentration in the cell extract was measured according to Bradford (1976) using the Coomassie protein assay reagent (Thermo Scientific, Waltham, MA USA, [34]). Protein concentration was calculated based on a calibration curve obtained with bovine serum albumin (BSA) standard solutions (Thermo Scientific). The glutathione concentration in the cell extracts was determined using the Glutathione Colorimetric Detection Kit (Thermo Fisher Invitrogen). Each measurement was performed in triplicate, and the means and standard deviations were then calculated. Intracellular glutathione was calculated in nmol per mg of protein in the cell extract (nmol/mg), per mg per ml of cell extract (mg/mL). For the latter process, cell disruption efficiency was considered; this was determined by plate counting before and after the glass bead treatment. The glutathione productivity was expressed as nmol of glutathione per gram of dry cell weight (nmol/g_DCW_). The quantification of inulinase activity was performed as described previously [35]. Briefly, the culture samples were centrifuged at 5000 × *g* at 4 °C for 10 min and the supernatant was collected. The mixture of 100 μL of supernatant and 900 µL of phosphate buffer (100 mM, pH 6.0) supplemented with 1.0% inulin was incubated at 50 °C for 15 min. Inactivation of the inulinase was immediately carried out by heating the reaction mixture at 100 °C for 10 min. The amount of fructose released was quantified by HPLC (Agilent Technologies, Santa Clara, CA, USA, 1200 series) on an Aminex HPX-87H column (300 mm × 7.8 mm, Biorad Laboratories, Hercules, CA, USA). Elution was performed at 40 °C at a flow rate of 0.6 mL/min using H_2_SO_4_ 5 mM solution as a mobile phase. Fructose was detected using a RID detector (Agilent Technologies, Santa Clara, CA, USA) set at 40 °C. This was then quantified by using standard solutions. One unit (U) of inulinase activity corresponded to 1 μmol of fructose released per minute.

## 3. Results and Discussion

The main purpose of this study was to evaluate *Y. lipolytica* as a producer of GSH. As a first step, the yeast was engineered with the aim of increasing GSH productivity. Then, different organic wastes were considered as sources of carbon and energy for both cell growth and GSH production. The last step consisted of the evaluation of GSH production from the organic wastes in the bioreactor.

### 3.1. Overexpression of Genes ylGSH1 and ylGSH2 

In order to identify the genes which encode γ-glutamylcysteine synthetase and glutathione synthase in *Y. lipolytica*, the *GSH1* and *GSH2* gene sequences from S*accharomyces cerevisiae* were used for BlastP analysis (sequences NP_012434.1 and NP_014593.1, respectively). The highest sequence identities were found for the genes *YALI0E30129p* and *YALI0C17831p* with values of 46% and 42%, respectively, suggesting that they correspond to the homologs of the genes *GSH1* and *GSH2* (hereafter, *ylGSH1* and *ylGSH2*; data not shown). With the aim of constructing *Y. lipolytica* strains with increased GSH productivity, these two genes were cloned under the control of the strong constitutive *TEF* promoter (pTEF) and expressed in the strain RIY145 (uracil auxotroph) or Po1d (leucine and uracil auxotroph) (Table 1). 

**Figure 1:**
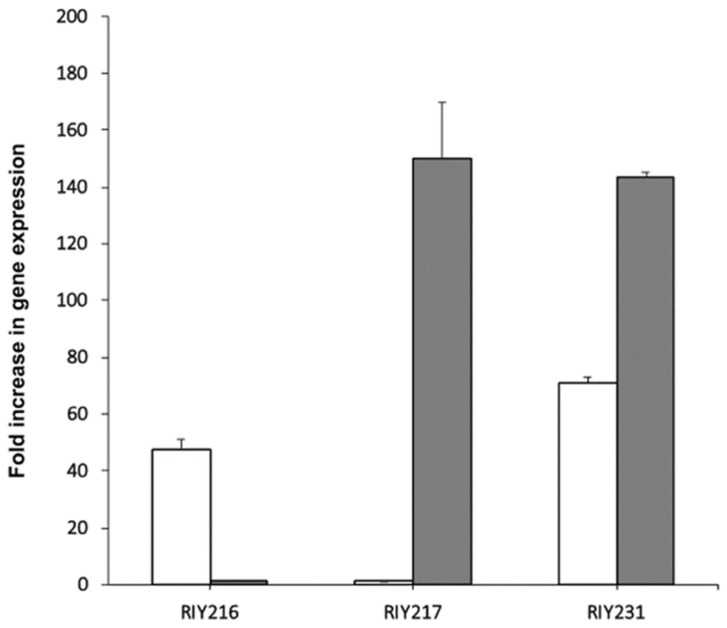
Transcriptional analysis of the genes ylGSH1 and ylGSH2 in strains RIY216 (p*TEF*-*ylGSH1*), RIY217 (p*TEF*-*ylGSH2*) and RIY231 (p*TEF*-*ylGSH1*-*ylGSH2*). Strains were grown in YPD for 24 h. Data are the mean and standard deviation of triplicate experiments. Values are expressed as the fold difference compared to the corresponding expression level in wild-type strain JMY2900. The gene *ylGSH1* is shown in white and *ylGSH2* in light grey.

**Figure 2:**
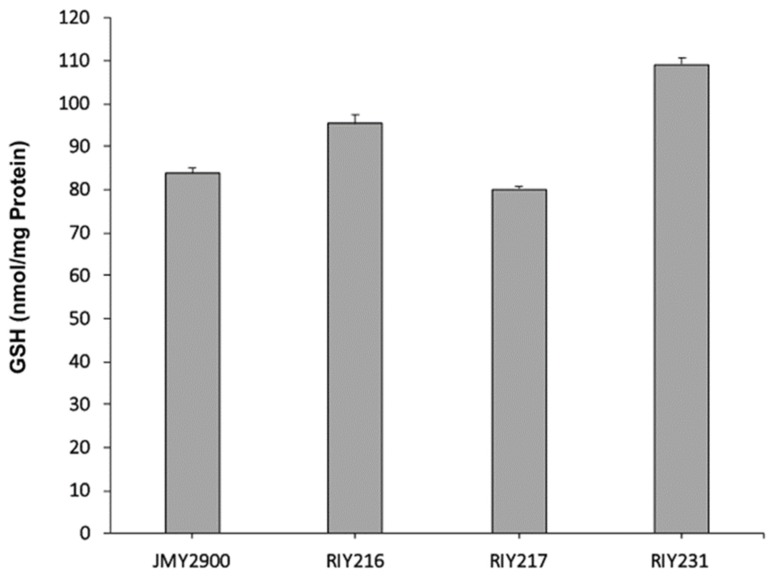
γ-l-glutamyl-l-cysteinyl-glycine (GSH) production of *Y. lipolytica* strains JMY2900 (WT), RIY216 (p*TEF*-*ylGSH1*), RIY217 (p*TEF*-*ylGSH*2) and RIY231 (p*TEF*-*ylGSH*1-*ylGSH*2) grown in YPD for 24 h. Data are the mean and standard deviation of three experiments. The glutathione titer value was expressed in nmol per mg of protein (nmol/mg protein).

In the resulting strains RIY216 (p*TEF-ylGSH1*) and RIY217 (p*TEF-ylGSH2*), the expression of the genes *ylGSH1* and *ylGSH2* were increased by 60 and 150-fold, respectively, as compared to the parental strain (Figure 1). In the strain RIY231 (p*TEF-ylGSH1-ylGSSH2*), the genes *ylGSH1* and *ylGSH2* were overexpressed by 71 and 143-fold, respectively (Figure 1). The three strains were then grown in YPD medium for 24 h, and the intracellular GSH content was determined. As shown in Figure 2, the overexpression of *ylGSH1* and *ylGSH2* alone or in combination had a different effect on the glutathione titer value. In strain RIY216 (p*TEF-ylGSH1*), the GSH titer value was increased by 14% (95 ± 2 nmol/mg protein) as compared to the wild-type strain (84 ± 1 nmol/mg protein). By contrast, the overexpression of *ylGSH2* did not increase the GSH production in the strain RIY217 (80 ± 1 nmol/mg protein). The overexpression of both *ylGSH1* and *ylGSH2* genes in the strain RIY231 yielded a 30% increase in the glutathione titer value (109 ± 1 nmol/mg protein). The feed-back inhibition of the γ-glutamylcysteine synthetase (encoded by *ylGSH1*) by GSH is a known mechanism of regulation [1]. Therefore, we investigated whether the expression of multi-copy *ylGSH1* in the strain RIY231 (p*TEF-ylGSH1-ylGSSH2*) would increase the GSH titer value. Unfortunately, in the resulting strains RIY390 (two copies) and RIY391 (three copies), no further increase of the GSH titer value was detected (data not shown).

### 3.2. Engineering Strain RIY231 for Carbon Catabolism

*Y. lipolytica* is known for its ability to catabolize uncommon substrates such as alkanes, triglycerides or fatty acids [7]. Unfortunately, these substrates are not well suited to bioreactor process operations. mainly due to their hydrophobic (water insoluble) nature. Therefore, several agro-industrial by-products which are better adapted to these processes were considered. More precisely, we focused on glycerol as a byproduct of the soap and biodiesel industry, starch as a byproduct of the potato transformation and bakery industries, lactose (whey) as a byproduct of the dairy industry, and inulin as a byproduct of the production of fructo-oligosaccharides (FOS) by the functional food industry. Although these carbon sources are potential interesting feedstocks, *Y. lipolytica* is unable to metabolize most of them naturally. Therefore, the specific genes described in Table 2 were constitutively overexpressed in strain RIY231 (p*TEF-ylGSH1-ylGSSH2)* to endow the yeast with the ability to metabolize these different carbon sources.

**Figure 3:**
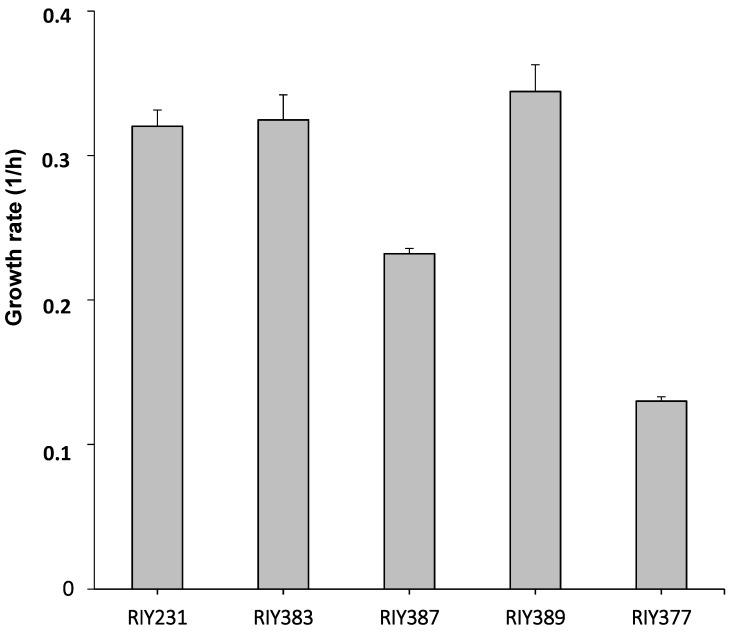
Growth rate of *Y. lipolytica* strains RIY231 (p*TEF*-*ylGSH*1-*ylGSH*2), RIY383 (p*TEF-ylGSH1-ylGSH2-GUT1-GUT2*), RIY387 (p*TEF-ylGSH1-ylGSH2-gAMY-αAMY*), RIY389 (p*TEF-ylGSH1-ylGSH2-INU1*), RIY377 (p*TEF-ylGSH1-ylGSH2-βGAL*) grown for 24 h in YNB medium supplemented with 10 g/L of glucose, glycerol, starch, inulin or lactose, respectively. Data are the mean and standard deviation of three experiments.

As a first characterization, the resulting strains were grown for 24 h in YNB medium containing 10 g/L of the different pure carbon sources, namely glycerol (YNBG), starch (YNBS), inulin (YNBI), lactose (YNBL) and glucose (YNBD). The growth rate of strains RIY383, RIY387, RIY389, RIY377 and RIY231, respectively, were then calculated. As shown in Figure 3, strains RIY383 (glycerol adapted) and RIY389 (inulin adapted) grew better (0.32 and 0.34 h^−1^, respectively) compared to strain RIY231 (p*TEF*-*ylGSH1*-*ylGSH2*) on glucose medium (0.32 h⁻^1^). By contrast, strains RIY387 (starch adapted) and RIY377 (lactose adapted) had a significantly lower growth rate (0.23 h^−1^ and 0.13 h^⁻1^, respectively). These cell growth values could be attributed to a weak ability of strain RIY387 and RIY377 to hydrolyze starch and lactose, respectively. 

**Figure 4:**
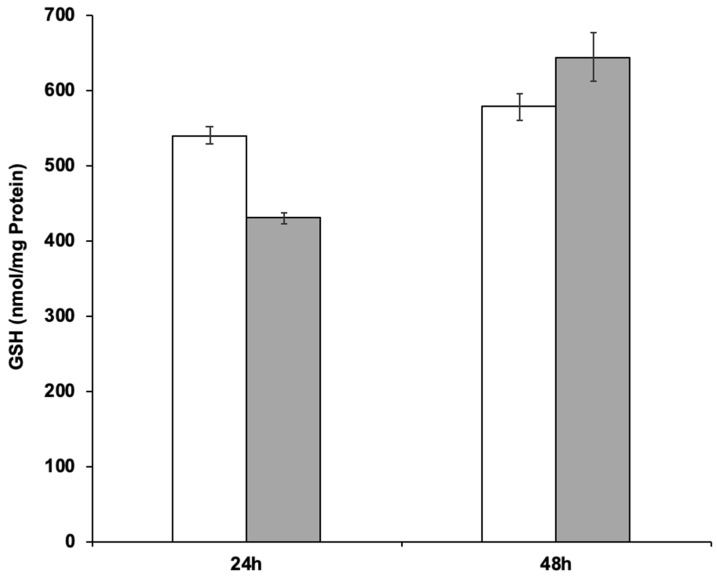
GSH production of *Y. lipolytica* strains RIY231 (p*TEF*-*ylGSH*1-*ylGSH*2), RIY383 (p*TEF-ylGSH1-ylGSH2-GUT1-GUT2*) and RIY389 (p*TEF-ylGSH1-ylGSH2-INU1*) grown for 24 h in YNB medium supplemented with 10 g/L of glucose, glycerol and inulin. The glutathione titer value was expressed in nmol per mg of protein (nmol/mg protein). Data are the mean and standard deviation of three experiments.

For strains RIY383 (glycerol adapted) and RIY389 (inulin adapted), the intracellular GSH content was also determined after 48 h of culture and compared to that obtained for the strain RIY231 grown on glucose medium (Figure 4). For strain RIY383, the GSH titer value was decreased by 36% as compared to the strain RIY231 (214 and 321 nmol/mg protein, respectively). By contrast, this was increased by 38% and 61% for the strain RIY389 as compared to strains RIY231 and RIY383, respectively (525 nmol/mg protein). To date, we have no hypothesis explaining the higher GSH productivity from fructose polymers, and no similar phenomenon has been reported in the literature to date. However, as inulin yielded the highest growth rate and GSH titer value, this carbon source was selected for further experiments. Inulin has already been reported to be a promising resource for the production of ethanol, single cell oils and other chemicals [8,36]. 

### 3.3. GSH Production from Inulin-Rich By-Products

Among the different carbon sources tested, inulin was the most suitable for GSH production. As a further characterization of the strain RIY389, the *INU1* expression level and inulinase activity were determined during cell growth in YNBI medium containing 10 g/L of pure inulin. qPCR measurements confirmed that the *INU1* gene was overexpressed (150-fold higher than the actin gene; data not shown). This high expression level correlates with high extracellular inulinase activity. This was maximal during the mid-exponential growth phase (after 14 h of growth, 300 U/mL) and then decreased to 125 U/mL at the end of the growth phase (after 22 h; data not shown). The inulinase activity of strain RIY389 was in the range obtained previously in yeasts, including *Y. lipolytica* [18,35].

As the purpose of the present research is to develop a cost-effective process to produce glutathione, raw extracts from different plants with a high inulin content were tested for their ability to support cell growth and GSH synthesis. They consisted of extracts from Jerusalem artichoke, chicory and blue agave. They were selected based on their utilization by the functional food industry for the production of fructo-oligosaccharides (FOS). As the FOS extraction process yield ranges between 0.6 and 0.8, it generates inulin-rich by-products [37]. Therefore, the ability of strain RIY389 to grow on these extracts was first investigated in YNB medium during shake flask cultures (with YNBIja, YNBc and YNBIa media, respectively). For the different extracts tested, cell growth was not significantly different (0.32 h^−1^ in average) compared to that obtained for pure inulin-based medium (0.31 h^−1^) (Table 3). The maximal biomass obtained after 48 h of culture was also similar (10.2 g_DCW_/L in average), except for medium containing Jerusalem artichoke extract, which yielded a lower biomass (6.5 g_DCW_/L). The GSH titer value and productivity were determined after 48 h of culture. The highest values were obtained for inulin extract from Jerusalem artichoke (707 nmol/mg protein and 358 µmol/g_DCW_, respectively; Table 3). For the other plant extracts tested, both GSH titer values and productivity were significantly lower (541 nmol/mg and 186 µmol/g_CDW_ on average). The lowest values were obtained for pure inulin (530 nmol/mg and 180 µmol /g_CDW_). 

**Figure 5:**
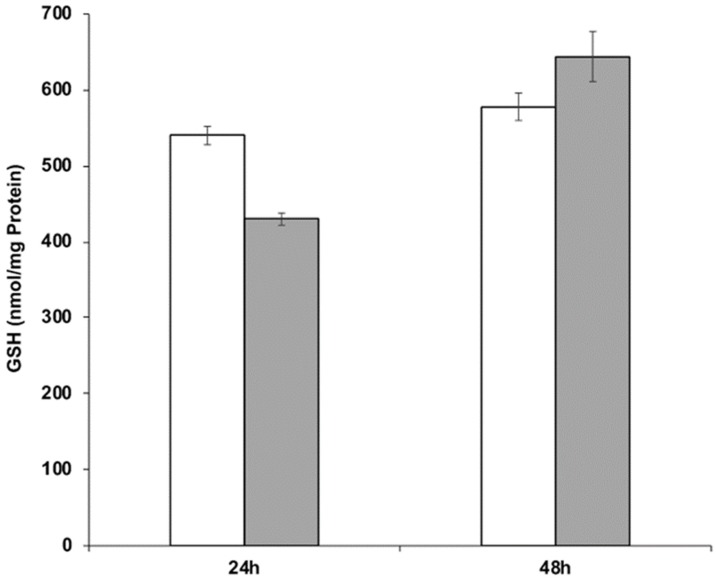
GSH production during the batch cultivation of the *Y. lipolytica* strain RIY389 (p*TEF-ylGSH1-ylGSH2-INU1*) in INU medium (white bars) and INUC medium (grey bars). The glutathione titer value was expressed in nmol per mg of protein (nmol/mg Protein). Data are the mean and standard deviation of two experiments.

The ability of strain RIY389 to produce GSH from Jerusalem artichoke extract was then investigated in the batch bioreactor. For that purpose, two media more consistent with industrial applications than YNBIja medium were considered, namely INU and INUC medium. INU medium only comprised Jerusalem artichoke extract while INUC medium contained (NH_4_)_2_SO_4_ and yeast extract in addition. Ammonium salt was used as an additional source of nitrogen, as it has been reported that Jerusalem artichoke has a low nitrogen content (1.2%; [38]). Yeast extract was used as a source of nutrients and minerals. The biomass was determined at the end of the growth phase (after 24 h) while the GSH content and productivity were determined after 48 h of culture, corresponding to their maximal value. As shown in Table 4 and Figure 5, the GSH titer values and productivity were higher in the INUC medium, with values equal to 644 nmol/mg protein and 510 µmol /g_CDW_, respectively. The results obtained with the strain RIY389 grown on inulin extract from Jerusalem artichoke are encouraging as compared to other published works. Indeed, with the mutant strain of *Y. lipolytica* obtained by N-nitrosoguanidine mutagenesis and *GSH2* overexpression, a glutathione titer value of 19.5 nmol/mg protein after 28 h of growth in YNB medium supplemented with glutamic acid, cysteine, and glycine was reported [39]. In *S. cerevisiae* with kinetically improved glutathione synthetase, the glutathione titer value was equal to 37.7 nmol/mg protein [40]. With the *S. cerevisiae* mutant overexpressing *GSH1* and its transcriptional activator *YAP1*, a glutathione titer value of 41.6 nmol/mg protein was obtained during ethanol-stat fed-batch cultivation [41]. For the methylotrophic yeast *Ogataea polymorpha*, with an overexpression of the *GSH2* gene and *MET4* encoding the central regulator for sulfur metabolism, the glutathione production after 96 h was equal to 837 nmol/mg protein in YNB medium containing 20 g/L of glucose. In the batch bioreactor, the glutathione titer value reached 420 nmol/mg protein after 71 h of cultivation [3]. In *S. cerevisiae* engineered to catabolize xylose, GSH was produced from lignocellulose-derived sugars with a titer value of 215.9 mg/L [4].

## 4. Conclusions

In this study, we aimed to develop *Y***.** *lipolytica* strains with an increased ability to produce glutathione from agro-industrial by-products. For that purpose, yl*GSH1* and yl*GSH2* encoding γ-glutamylcysteine synthetase and GSH synthetase were first expressed under the control of a strong pTEF promoter. In a second step, the resulting strain RIY389 was further genetically optimized to catabolize different raw carbon sources. From this, inulin extract from Jerusalem artichoke yielded higher glutathione titer values and productivity in a batch bioreactor, at 644 nmol/mg protein and 510 µmol/g_CDW_, respectively, after 48 h of culture_._ These values are the highest ever reported for *Y. lipolytica* and are competitive as compared to those of other engineered organisms such as *O. polymorpha* or *S. cerevisiae*.

## Figures and Tables

**Table 1:** Plasmids and *Y. lipolytica* used in this study.

Strains	Genotype-Plasmid	Source/Reference
*E. coli*		
DH5α	Δ(*lac*ZYA-*arg*F)U169 *rec*A1 *end*A1 *hsd*R17(rK^-^ mK^+^) *pho*A *sup*E44 λ^–^ *thi*-1 *gyr*A96 *rel*A1 F^–^ φ80lacZΔM15	Promega
RIE102	RIP102, JMP62 derivative, pTEF-*FUM*, *URA3*ex	[28]
RIE103	RIP103, JMP62 derivative, pTEF-*FUM*, *LEU2*ex	Lab stock
RIE153	RIP153, pGEMTeasy-m*GSH*1	This work
RIE155	RIP155, pGEMTeasy-m*GSH*2	This work
RIE161	RIP161, pGEMTeasy-m*GSH*1 (w/o *BamH*I)	This work
RIE162	RIP162, pGEMTeasy-m*GSH2* (w/o *Avr*II)	This work
RIE210	RIP210, JMP62 derivative, pTEF-m*GSH1 LEU2*ex	This work, expression vector
RIE211	RIP211, JMP62 derivative, pTEF-m*GSH1 URA3*ex	This work, expression vector
RIE212	RIP212, JMP62 derivative, pTEF-m*GSH2 LEU2*ex	This work, expression vector
RIE213	RIP213, JMP62 derivative, pTEF-m*GSH2 URA3*ex	This work, expression vector
RIE282	RIP282, JMP62 derivative, pTEF-*INU1*, *LEU2*ex	[29], expression vector
RIE287	RIP287, JMP62 derivative, pTEF-*glucoAMY*, *LEU2*ex	[14], expression vector
RIE288	RIP288, JMP62 derivative, pTEF-*alphaAMY*, *LEU2*ex	[14], expression vector
JME2792	JMP2792, JMP62 derivative, pTEF-*GUT1, URA3*ex	[30], expression vector
FCE015	FCP015, JMP62 derivative, pTEF-*GUT2, URA3*ex	[30], expression vector
RIE196	RIP196, pINA1311-pHP4d-PirI-A.*ory*Gal	[31], expression vector
RIE112	RIP112 pUB4-CRE (*Cre, hph*)	[27], Cre recombinase
*Y. lipolytica*		
Po1d	*ura3-303 leu2-270 xpr2-322* MATA	[24]
JMY2900	Po1d, *URA*3, *LEU*2	[14]
RIY145	Po1d, *LEU2*	[32]
RIY176	Po1d Δ EYK ura-leu-	[32]
RIY216	RIY145 pTEF-*GSH1*, *URA3*ex	This work
RIY217	RIY145 pTEF-*GSH2*, *URA3*ex	This work
RIY231	Po1d, Δ*eyk1*-pTEF-*GSH1-GSH2*, *URA3*ex, *LEU2*ex	This work
RIY377	Po1d, Δ*eyk1*-pTEF-*GSH1-GSH2- βGAL*, *URA3*ex, *LEU2*ex	This work
RIY383	Po1d, Δ*eyk1*-pTEF-*GSH1-GSH2-GUT1-GUT2*, *LEU2*ex, *URA3*ex	This work
RIY387	Po1d, Δ*eyk1*-pTEF-*GSH1-GSH2-glucoAMY-αAMY*	This work
RIY389	Po1d, Δ*eyk1*-pTEF-*GSH1-GSH2-INU1*, *LEU2*ex, *URA3*ex	This work
RIY390	Po1d, Δ*eyk1*-pTEF-*GSH1-GSH2-GSH1*	This work
RIY391	Po1d, Δ*eyk1*-pTEF-*GSH1-GSH2-GSH1-GSH1*	This work

**Table 2:** Genes overexpressed for raw carbon metabolism.

Genebank ID.	Name	Encoding Enzyme	Template DNA Source	Carbon Source	Reference
NP_414878.1	*β-GAL*	*β-*galactosidase	*E. coli*	Lactose	[31]
YALI0F00484g	*GUT1*	Glycerol kinase	*Y. lipolytica*	Glycerol	[30]
YALI0B13970g	*GUT2*	Glycerol -3P dehydrogenase	*Y. lipolytica*	Glycerol	[30]
Synthetic gene	*α-AMY*	alpha-amylase	*Oryza sativa*	Starch	[14]
XM_001390493	*g-AMY*	gluco-amylase	*A. niger*	Starch	[14]
X57202.1	*INU1*	Inulinase	*K. marxianus*	Inulin	[18]

**Table 3:** Dynamics of GSH and biomass production of strain RIY389 (pTEF-ylGSH1-ylGSH2-INU1) during culture in a shake flask in YNB medium supplemented with glucose, fructose or different inulin extracts. Growth rate was calculated between 4 and 22 h of culture; GSH and biomass values were determined after 48 h of culture. Data are the mean and standard deviation of triplicate experiments.

Inulin	Growth Rate (h^−1^)	Biomass (g_DCW_/L)	GSH (nmol/mg Protein)	Inulin Content (%)	Productivity (µmol/g_DCW_)
Pure	0.33 ± 0.01	10.4 ± 0.16	530 ± 63	100	180 ± 22
Jerusalem artichoke	0.36 ± 0.01	6.5 ± 0.09	707 ± 73	61	358 ± 30
Chicory	0.33 ± 0.01	9.6 ± 0.19	544 ± 63	90	184 ± 20
Blue agave	0.32 ± 0.01	9.0 ± 0.22	550 ± 90	92	160 ± 6
Glucose	0.32 ± 0.01	10.1 ± 0.07	277 ± 80	-	106 ± 4
Fructose	0.31 ± 0.02	9.3 ± 0.15	356 ± 30	-	136 ± 12

**Table 4:** Dynamics of GSH and biomass production during the batch cultivation of strain RIY389 (p*TEF*-*ylGSH1-ylGSH2-INU1*) in INU and INUC. Biomass was given after 24 h; GSH and productivity were determined after 48 h of culture. Data are the mean and standard deviation of two experiments.

Medium	Biomass (g_DCW_/L)	GSH (nmol/mg Protein)	GSH (mg/L)	Productivity (µmol/g_CDW_)
INU	3.3 ± 0.5	578 ± 18	310 ± 31	306 ± 32
INUC	4.0 ± 0.2	644 ± 33	626 ± 65	510 ± 32

## Supplementary Materials

The following are available online at https://www.mdpi.com/2076-2607/8/4/611/s1, Table S1: Primers used in the study.
