# Supplementary material for: Engineering Yarrowia lipolytica for the Synthesis of Glutathione from Organic By-Products
Source: Microorganisms. 2020 Apr 23;8(4):611. doi: 10.3390/microorganisms8040611 (PMC7232331; doi:10.3390/microorganisms8040611)
Supplement: Supplementary file 1 [file microorganisms-08-00611-s001.pdf]

## Supplementary Information

# Engineering *Yarrowia lipolytica* for the Synthesis of Glutathione from Organic By-Products

Diem T. H. Do, Patrick Fickers*

Microbial Processes and Interactions, TERRA Teaching and Research Center, University of Liège - Gembloux Agro-Bio Tech, Av. De la Faculté, 2B, 5030 Gembloux, Belgium

*Corresponding author: pfickers@ULiege.be; Tel.: +32 81 822 814

Table 1. Primers used in the study.

| Code | Primer name | Primer sequence 5'-3' | Modification/ utilization |
|---|---|---|---|
| N1 | GSH1-Fo | CACTGGATCCATGGGTCTCCTGTCGCT | *BamH*I added |
| N2 | GSH1-Rev | GCGCCTAGGCTACTCCTTCTCGTACTCAAAACC | *Avr*II added |
| N3 | GSH1-Bfo | CGCTACCTGGATTCGAAACTTCATCAG | Mutagenesis |
| N4 | GSH1-Brev | CTGATGAAGTTTCGAATCCAGGTAGCG | Mutagenesis |
| N5 | GSH2-Fo | GCTGGATCCATGACGTTCCAAGAGAAAATCAAAG | *BamH*I added |
| N6 | GSH2-Rev | CTTCCTAGGCTAATCCACAAGGTAAACACCATC | *Avr*II added |
| N7 | GSH2-Arev | GAACAGACCTGGGGTCAGGGG | Mutagenesis |
| N8 | GSH2-Afo | CCCTGACCCTGGGTCTGTTCC | Mutagenesis |
| N9 | qPCR-GSH1f | TGACTTCGACGACATTCTGC | RT-PCR GSH1 |
| N10 | qPCR-GSH1r | CACCCTTGGGCTCGTAATAA | RT-PCR GSH1 |
| N11 | qPCR-GSH2f | GTGTTCGACGAGAGCATTGA | RT-PCR GSH2 |
| N12 | qPCR-GSH2r | ATGTAGTCGGACCGGAACAG | RT-PCR GSH2 |
| N13 | LoxP-FO | GCATACATTATACGAAGTTATTCTGAATTC | Auxotrophic marker verification |
| N14 | LoxR-Rev | GGGTAATTATCGCTTCGGATA | Auxotrophic marker verification |
| N15 | pTEF-Fo | GGACCCAACCCCGGCG | Gene integration verification |
| N16 | INU1-MFo | TGGCATTTGTACTACCAGTACAACCCAG | - |
| N17 | INU1-MRev | GTACCGTTGAAGTCACCAACAAAGTATTG | - |
| N18 | alpha-AMY-LqFo | TAACCCTGACACTGGAGCTG | RT-PCR α-AMY |
| N19 | alpha-AMY-RqREV | CAGATCTCAGCAACGGCAAA | RT-PCR α-AMY |
| N20 | gluco-AMY-LqFo | CGCTAACAACCGACGAAACT | RT-PCR g-AMY |
| N21 | gluco-AMY-Rqrev | TCTTAGAGGCGGTAGCAGTG | RT-PCR g-AMY |
| N22 | Inu1-LqFo | GCTCCAACTTCAGAGACCCT | RT-PCR INU1 |
| N23 | Inu1-RqRev | CGGAGGAGTTCGAAGAGTCA | RT-PCR INU1 |
| N24 | GUT1-L-qPCR | CCCTGTCCACCTACTTTGCC | RT-PCR GUT1 |
| N25 | GUT1-R-qPCR | TTGGAGGTGTCGGTGATGTG | RT-PCR GUT1 |
| N26 | GUT2-P-L-qPCR | CAGCAAGAGAAGCAACGGTGA | RT-PCR GUT2 |
| N27 | GUT2-T-R-qPCR | CCAAGTTCGGTCTCAAGCCTG | RT-PCR GUT2 |
